# Derivation of Human Differential Photoreceptor-like Cells from the Iris by Defined Combinations of *CRX*, *RX* and *NEUROD*

**DOI:** 10.1371/journal.pone.0035611

**Published:** 2012-04-25

**Authors:** Yuko Seko, Noriyuki Azuma, Makoto Kaneda, Kei Nakatani, Yoshitaka Miyagawa, Yuuki Noshiro, Reiko Kurokawa, Hideyuki Okano, Akihiro Umezawa

**Affiliations:** 1 Department of Reproductive Biology, Center for Regenerative Medicine, National Institute for Child Health and Development, Okura, Setagaya, Japan; 2 Sensory Functions Section, Research Institute, National Rehabilitation Center for Persons with Disabilities, Tokorozawa, Japan; 3 Department of Ophthalmology, National Center for Child Health and Development, Setagaya, Japan; 4 Department of Physiology, Keio University School of Medicine, Shinanomachi, Japan; 5 Institute of Biological Science, Tsukuba University, Ten-noudai, Japan; Sanford-Burnham Medical Research Institute, United States of America

## Abstract

Examples of direct differentiation by defined transcription factors have been provided for beta-cells, cardiomyocytes and neurons. In the human visual system, there are four kinds of photoreceptors in the retina. Neural retina and iris-pigmented epithelium (IPE) share a common developmental origin, leading us to test whether human iris cells could differentiate to retinal neurons. We here define the transcription factor combinations that can determine human photoreceptor cell fate. Expression of rhodopsin, blue opsin and green/red opsin in induced photoreceptor cells were dependent on combinations of transcription factors: A combination of *CRX* and *NEUROD* induced rhodopsin and blue opsin, but did not induce green opsin; a combination of *CRX* and *RX* induced blue opsin and green/red opsin, but did not induce rhodopsin. Phototransduction-related genes as well as opsin genes were up-regulated in those cells. Functional analysis; i.e. patch clamp recordings, clearly revealed that generated photoreceptor cells, induced by *CRX*, *RX* and *NEUROD*, responded to light. The response was an inward current instead of the typical outward current. These data suggest that photosensitive photoreceptor cells can be generated by combinations of transcription factors. The combination of *CRX* and *RX* generate immature photoreceptors: and additional *NEUROD* promotes maturation. These findings contribute substantially to a major advance toward eventual cell-based therapy for retinal degenerative diseases.

## Introduction

The possibility of redirecting cell differentiation by overexpression of genes was suggested by Weintraub with the identification of the “master gene,” *MyoD* [1]. The process was thought to involve reversion to a less differentiated state, a kind of de-differentiation, before the new cell type is formed. Another process has since been introduced, the concept of “direct conversion” or “direct reprogramming” without de-differentiation. This process is thought to be direct lineage switching [2] rather than lineage switching back to a branch point and out again in a different direction. “Direct conversion” has been shown in beta-cells, cardiomyocytes and neurons: A specific combination of three transcription factors (Ngn3, Pdx1 and MafA) reprogram differentiated pancreatic exocrine cells in adult mice into cells that closely resemble beta cells [3]; a combination of three factors (Gata4, Tbx5 and Baf60c) induces non-cardiac mesoderm to differentiate directly into contractile cardiomyocytes [4]; and a combination of three factors (Ascl1, Brn2 and Myt1l) converts mouse fibroblasts into functional neurons [5]. In this study, we employed the strategy of “direct reprogramming” to generate retinal photoreceptor cells from human somatic cells.

Several retinal diseases, including retinitis pigmentosa, age-related macular degeneration and cone dystrophy, lead to loss of vision due to loss of photoreceptors and retinal pigment epithelium (RPE). Gene therapy has been implicated for Leber's congenital amaurosis [6]. Another promising therapeutic strategy is to transplant functional photoreceptor cells and retinal pigment epithelial cells. Sheets of human fetal neural retina with retinal pigment epithelium [7] and ES cell-derived photoreceptors [8] have been implicated for use as sources for photoreceptor cells. And human ES cell-derived RPE has recently been implicated to patients with macular degeneration [9]. However, the use of human embryos faces ethical controversies that prevent the widespread applications of human fetal tissues and human ES cells. A way to circumvent these issues is to induce photoreceptor-specific phenotypes by direct reprogramming of somatic cells of the patients. During vertebrate eye development, the inner layer of the optic cup differentiates into the neural retina and iris-pigmented epithelium (IPE). This common developmental origin led us to test whether iris cells could transdifferentiate to retinal neurons and thus be a candidate source of cells for transplantation. We here define the combinations of transcription factors that induce light responsive photoreceptor-like cells in humans.

## Results

### Cultivation of iris-derived cells

**Figure 1:**
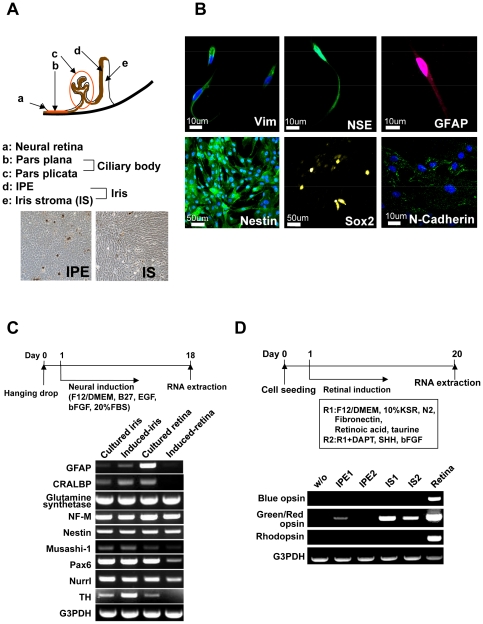
Retinal glia- and retinal progenitor-like phenotypes in iris cells. (A) Scheme of cell sources in the iris and ciliary body. (B) Immunocytochemical analysis of iris cells. Iris cells are immunocytochemically positive for glial cell marker (GFAP), and neural stem cell markers (Nestin (green), Sox2 (yellow) and N-Cadherin (green)). Nuclei were stained with DAPI (blue) with vimentin, nestin and N-cadherin. (C) Expression of neuron-related genes after neural induction. RT-PCR analysis indicates that iris cells expressed glial cell markers (GFAP, CRALBP and glutamine synthetase), and neural stem cell markers (Nestin, Musashi-1 and Pax6). By the “hanging-drop” method coupled with the B27 medium, rhodopsin was not induced. In this illustration, “Induced” indicates “cells at an induced state by the hanging-drop method coupled with the B27 medium” and “Retina” indicates retina-derived cells at passage 3. (D) Expression of the opsin genes after retinal induction. In this illustration, “w/o” indicates iris-stromal cells without any induction. “IPE” and “IS” indicate “iris pigment epithelial cells” and “iris-stromal cells”, respectively, that were induced by exogeneously added chemicals and growth factors as indicated. By retinal induction with the R1 medium, green/red opsin was up-regulated significantly in iris-stromal cells, but blue opsin or rhodopsin was not up-regulated.

The iris pieces were cut into smaller pieces and served as explants culture. Cells derived from the iris pieces are designated as “iris cells”. Iris pigment epithelial cells (IPE cells) were isolated from iris tissues using dispase and trypsin. Residual iris pieces after removal of IPE were cut into smaller pieces and served as explant culture. Outgrowing cells from the explant cultures were designated as “iris-stromal (IS) cells” (Fig. 1A). Ciliary epithelial cells were isolated from pars plana and pars plicata in the same manner as IPE cells. We then performed Southern blot analysis and nucleotide sequencing to investigate whether the RB gene was deleted or mutated, because some irreversibly de-identified iris-derived cells were from the patients with retinoblastoma. Southern blot analysis revealed that the RB gene was not deleted or rearranged in any of the iris-derived cells examined (Fig. S1). Sequencing analysis revealed that cDNAs of the RB gene did not have deletions or mutations at the nucleotide level.

### Cultured iris cells show phenotypes of retinal glia and progenitor

Iris cells were immunocytochemically positive for glial cell- and neural stem cell-markers (Fig. 1A, 1B). RT-PCR analysis revealed that these cells expressed markers for glial cells and neural stem cells, indicating that the iris has common features with the retinal glia (Fig. 1C). After neural induction with the B27 medium, rhodopsin was not induced (Fig. 1C). After retinal induction with the R1 medium, green/red opsin was up-regulated significantly but blue opsin and rhodopsin were not up-regulated (Fig. 1D).

### Iris cells are induced into a rod- or cone-specific phenotype by defined transcription factors

We selected six genes, *SIX3*, *PAX6*, *RX*, *CRX*, *NRL*, and *NEUROD*, as candidate factors that may contribute to induce photoreceptor-specific phenotypes in iris cells, on the basis that such factors play pivotal roles in the development of photoreceptors. Iris cells were transfected with these genes and were examined for inducible expression of photoreceptor-specific genes in those cells. Transduction of a single gene for *SIX3*, *PAX6*, *RX*, *CRX*, *NRL*, or *NEUROD* induced neither rod- nor cone-specific phenotypes in iris cells, but the six genes together up-regulated blue opsin and rhodopsin (Fig. S2). To determine which of the six candidates were critical, we tested the effect of withdrawal of individual factors from the pool of transduced candidate genes on expression of the opsin genes. We identified two genes, *NEUROD* and *CRX*, which were essential for photoreceptor induction; individual withdrawal of *NEUROD* resulted in loss of expression of rhodopsin and withdrawal of *CRX* resulted in loss of blue opsin.

**Figure 2:**
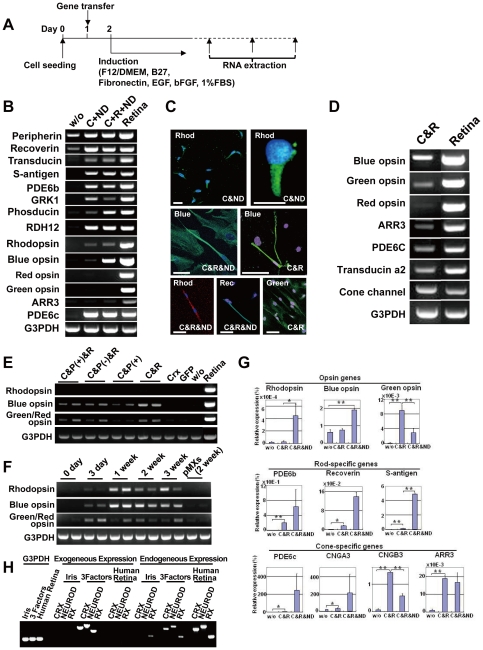
Induction of rod- or cone-specific phenotypes in human iris cells by the defined transcription factors. (A) Protocol to induce rod- or cone-specific phenotypes in human iris cells by the defined transcription factors. (B) Expression of rod-specific genes in iris cells after transduction of the combination of *CRX* and *NEUROD* or the combination of *CRX*, *RX*, and *NEUROD*. The combination of only two genes, *CRX* and *NEUROD* induced expression of rhodopsin, i.e. rod-photoreceptor specific opsin. Addition of *RX* to *CRX* and *NEUROD* enhanced blue opsin expression. “w/o”: cultured iris-derived cells without gene transfer as a negative control; “Retina”: human retinal tissue as a positive control. (C) Immunocytochemistry using antibodies to blue opsin (green), green/red opsin (green), rhodopsin (green or red) and recoverin (green). Nuclei were stained with DAPI (blue). Experiments were performed at two weeks after infection. “Blue”: blue opsin; “Green”: green/red opsin; “Rhod”: rhodopsin; “Rec”: recoverin. Scale bars represent 10 µm in the upper left panel and 50 µm in the other panels. (D) Transduction of cone-specific genes in iris cells. Cone-specific phenotypes were induced by the transcription factors, i.e., the combination of *CRX* and *RX*. The combination of *CRX* and *RX* induced other cone-specific genes in addition to the blue opsin, green opsin and red opsin genes. (E) Effect of *PAX6* (+5a) on expression of opsin genes. “Retina”: human retinal tissue as a positive control; “w/o”: cultured iris-derived cells without gene transfer as a negative control; “GFP”: cultured iris-derived cells after transduction of GFP genes as another negative control. (F) Time course of gene expression after transduction of *RX*, *CRX* and *NEUROD*. Expression of the rhodopsin and blue opsin genes increased one week after transduction and then remained unchanged at a later stage. Expression of the green/red opsin gene reached a maximum level three days after infection. Each independent experiment was performed in duplicate as shown in the panel. (G) Quantitative RT-PCR results for rhodopsin, blue opsin, green opsin, PDE6b, recoverin, S-antigen, PDE6c, cone channel A3, cone channel B3 and arrestin3 (ARR3). Vertical axis indicates expression levels of each gene (%) in the indicated cells, relative to human retinal tissues. *p<0.05 and **p<0.005 (Welch's t-test). (H) RT-PCR analysis of the exogenous and endogenous genes in induced retinal cells. Expression of the *CRX, NEUROD* and *RX* genes in the iris cells and transgene-induced cells was analyzed by RT-PCR, using the exogenous and endogenous gene-specific primers (Table S1). Human retina served a control for the endogenous genes. Equal amounts of RNAs were examined by expression of the G3PDH gene.

Then, we tested the combination of only two genes, CRX and NEUROD (Fig. 2A, 2B). The combination of *CRX* and *NEUROD* induced rod photoreceptor specific genes including rhodopsin and other phototransduction genes. After transduction of *CRX* and *NEUROD*, immunostaining showed that 38% of total cells were rhodopsin-positive cells (3,750 cells) (Fig. 2C, Fig. S3). However, this combination did not induce the red opsin gene. Addition of *RX* to the combination of *CRX* and *NEUROD* augmented blue opsin expression (Fig. 2B). After transduction with *CRX*, *RX* and *NEUROD*, rhodopsin-positive, blue opsin-positive and green/red opsin-positive cells were 29% (per 954 cells), 37% (per 235 cells) and 25% (per 193 cells) of total cells, respectively, by immunostaining. Hybrid photoreceptor cells were also detected by double-staining immunocytochemistry (Fig. S4). We then investigated combinations of transcription factors that induce specific types of photoreceptor cells. A combinational approach showed that combination of *CRX* and *RX* was sufficient to induce green/red opsin and other cone-specific genes (Fig. 2D, Fig. S2). *PAX6*(+5a) did not influence cone-related gene induction (Fig. 2E). Expression levels of rhodopsin and blue opsin reached a maximum level by one week after gene transduction and remained unchanged up to 3 weeks. Expression of green/red opsin reached a maximum level 3 days after gene transduction (Fig. 2F). Expression levels of opsin- and phototransduction-related genes were quantitated (Fig. 2G). *NEUROD* significantly decreased expression of the cone-specific genes, i.e. genes for green opsin and cone channel B3 (CNGB3) in human iris cells (p<0.005). On the other hand, it was clearly demonstrated that expression of rhodopsin and S-antigen, which are specifically expressed in rod photoreceptors, were much higher in *CRX*, *RX* and *NEUROD*-infected cells than in *CRX* and *RX*-infected cells (rhodopsin, p<0.05; S-antigen, p<0.005, Welch's t-test). Ultrastructural analysis revealed a cilia-associated structure, i.e. centriole, surrounded by mitochondria (Fig. S5).

### Inhibition of factors by small interfering RNA (siRNA)

We performed RT-PCR to investigate if the transgenes continued to be expressed in the generated retinal cells (Fig. 2H, Table S1). The exogenous factors (transgenes) were clearly detected in induced retinal cells. Interestingly, the corresponding endogenous genes intiated expression in the induced retinal cells, similar to what is found in iPS cells. We then suppressed the *CRX* and *NEUROD* genes by siRNA (Fig. S6) to investigate the involvement of the genes in photoreceptor differentiation. Expression of the photoreceptor-specific/associated genes (blue opsin, s-antigen and recoverin) decreased significantly in siCRX and siNEUROD-transfected cells, compared to cells treated with control siRNA, suggesting that CRX and NEUROD are necessary for photoreceptor conversion.

### Derivation of photoreceptor-like cells from IPE and IS cells

**Figure 3:**
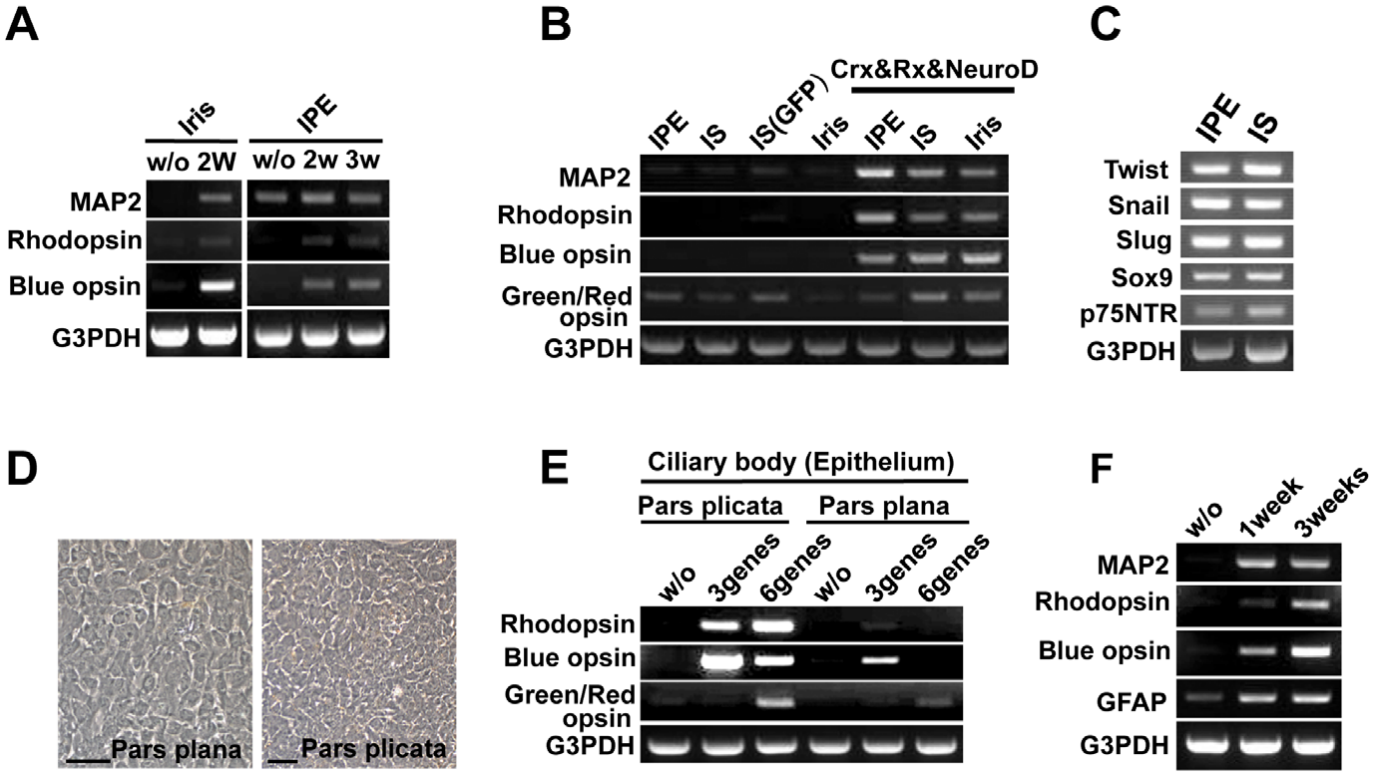
Induction of opsin genes in human iris-derived cells, ciliary epithelial cells and retina-derived cells by the retroviral infection of all the 6 genes and genes for *RX*, *CRX* and *NEUROD*. (A) RT-PCR analysis for genes of MAP2, rhodopsin, blue opsin and G3PDH in two kinds of iris cells after gene transfer of all the six genes. All six genes were infected into two kinds of iris cells: IPE and stromal cells derived from the peripheral iris, and purely isolated IPE cells. In both cell types, rhodopsin and blue opsin genes were up-regulated. “w/o”: cultured iris-derived cells without gene transfer as a negative control. (B) Expression of the rhodopsin and blue opsin genes started two weeks after infection. “IS”, “IPE” and “Iris (central)” indicate “iris-stromal cells”, “iris pigment epithelial cells”, and “central iris cells”, respectively. “IS (GFP)” is “iris-stromal cells infected with the GFP gene”. In all kinds of iris cells, transduction of the three genes, that are *RX*, *CRX* and *NEUROD*, enhanced expression of rhodopsin, blue opsin and green/red opsin. (C) RT-PCR analysis for genes for genes of neural crest-related markers in two kinds of iris-derived cells: iris stromal cells and iris pigmented epithelial cells. (D) Phase-contrast photomicrograph of ciliary epithelial cells from pars plana (left) and pars plicata (right). (E) RT-PCR analysis for genes of rhodopsin, blue opsin, green/red opsin and G3PDH in human ciliary epithelium (pars plicata and pars plana) after gene transfer of either all the six genes (*SIX3*, *PAX6*, *RX*, *CRX*, *NEUROD*, *NRL*) or three genes (*CRX*, *RX* and *NEUROD*). (F) RT-PCR analysis for the MAP2, rhodopsin, blue opsin, GFAP and G3PDH genes in retina-derived cells after transfer of all six genes. “w/o”: Retina-derived cells without gene transfer 21 days after the start of cultivation. Genes for MAP2, rhodopsin, and blue opsin started to be expressed after the gene transfer.

To investigate photoreceptor cell differentiation from other cell types, we isolated IPE and IS cells from iris tissues. Both cell types began to express opsin genes after transduction of *CRX*, *RX* and *NEUROD* genes (Fig. 3A, 3B). To determine if IPE and IS cells originated from neural ectoderm and neural crest cells, we investigated expression of neural crest marker genes. IPE and IS expressed these neural crest markers at high levels (Fig. 3C). These findings indicate that IS cells derived from neural crest cells, as well as IPE cells, could differentiate into photoreceptor-like cells. We also isolated ciliary epithelial cells from pars plicata and pars plana (Fig. 1A, 3D). Ciliary epithelial cells from pars plicata expressed rhodopsin, blue opsin, and green/red opsin at a high level after transduction with three genes (*CRX*, *RX* and *NEUROD*) or all six genes together (Fig. 3E). Retina-derived Müller glial cells expressed opsin genes after transduction of all genes (Fig. 3F).

### Induced photoreceptor-like cells are photoresponsive in vitro

**Figure 4:**
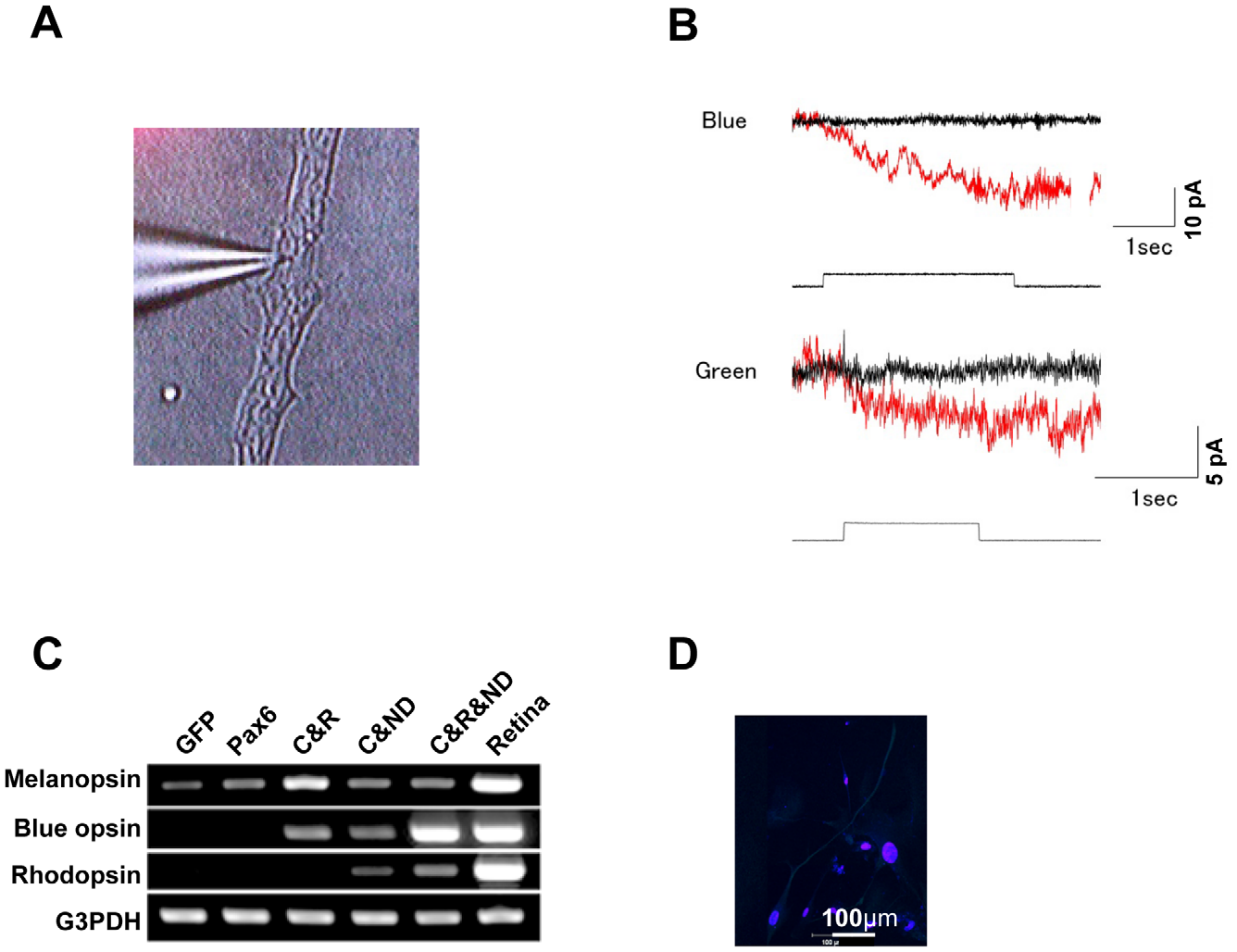
Electrophysiological analysis of the induced photoreceptor-like cells. (A) Recording electrode patched onto infected cells. (B) Responses to blue light (upper panels) or green light (lower panels) in infected cells (red) and non-infected cells (black). The light onset for transfected cells and non-transfected cells had the same timing. The square under the current trace is a timing and duration of light stimulation for transfected cells. The longer light stimulation was given to non-infected cells to rule out any possible artifact. Holding potential was −40 mV. Larger baseline noise in the infected cells probably reflects the channel activities. (C) RT-PCR analysis for genes of melanopsin, rhodopsin, blue opsin and G3PDH in iris cells after gene transfer. Cells were infected with retroviruses carrying the genes for GFP, *PAX6* (+5a) (Pax6), *CRX* & *RX* (C&R), *CRX* & *NEUROD* (C&ND) and *CRX* & *RX* & *NEUROD* (C&R&ND). “Human retina”: human retinal tissue, as a positive control. (D) Immunocytochemistry for melanopsin in iris-derived cells after transduction of *CRX*, *RX* and *NEUROD*. Nuclei were stained with DAPI (blue).

Light stimulation was applied to *CRX*, *RX* and *NEUROD*-infected human iris-derived cells because these infected cells showed the most photoreceptor-like phenotypes by RT-PCR and immunocytochemistry. Both blue and green light stimulation produced inward current (Fig. 4A, 4B). Inward current continued to flow after the offset of light stimulation but only four cells showed partial or complete recovery within 60 sec after the cessation of light stimuli (n = 9), presumably reflecting the limited expression or absence of inactivation machinery. Inward current to blue light stimulation was observed in three out of four cells and inward current to green light stimulation was observed in six out of six cells. Light stimulation to non-infected control cells (blue, n = 2; green, n = 2) did not produce any inward current. These results indicate that the combination of *CRX*, *RX* and *NEUROD* transforms human iris-derived cells into photoresponsive photoreceptor-like cells in vitro, although the typical outward current of photoreceptor cells could not be detected. Since the light-induced inward current seemed to be mediated by melanopsin-associated phototransduction, we investigated expression of melanopsin by RT-PCR and immunocytochemistry. *CRX*, *RX* and *NEUROD*-infected iris-derived cells expressed melanopsin (Fig. 4C, 4D), suggesting a larger contribution of melanopsin-associated inward current.

## Discussion

This is the first report that functional photosensitive photoreceptor-like cells can be induced from human somatic cells. The present study shows that rod- and cone-photoreceptor-specific phenotypes were induced by transduction of a combination of *CRX*, *RX* and *NEUROD* genes, and that those cells responded to light electrophysiologically. In the retina, rod- and cone- photoreceptors convert light information to electrical signals that are relayed to the brain through several interneurons. In the present study, a combination of *CRX*, *RX* and *NEUROD* induced all of the opsin genes: blue opsin, green/red opsin and rhodopsin (Table S2). On the other hand, a combination of *CRX* and *RX* induced only cone-specific opsin, and additional transduction of *NEUROD* up-regulated rod-specific opsin and rod-specific phototransduction related genes.

Rod photoreceptor generation from iris cells required *NEUROD* in our study. NeuroD is a regulator of both rod photoreceptors [10], [11] and cone photoreceptors [12] during mouse development. NeuroD overexpression increases amacrine cells and rod photoreceptors, reduces bipolar cells, and inhibits formation of Müller glia. It has been known since the early 1960s that there is a defined sequence in formation of retinal neurons, which is largely conserved across vertebrates: Cone photoreceptors are generated during early stages of development, and most rod photoreceptors are generated in the latter half of the period of retinogenesis [13]. Similarly, cone photoreceptors are generated at the early stages during ES cell differentiation and rod photoreceptors are generated at a later stage. The present study and these previous reports suggest that NeuroD may work downstream to regulate the development of rod-photoreceptors. NeuroD generally functions in a cell cycle-specific manner, and promotes cell cycle exit [11]. Rod formation may thus be mediated via cessation of cell cycle by NeuroD at the later stage.

It has been hypothesized that retinal stem cells can be found in the ciliary body [14], postnatal retina, and the iris [15]. Pure populations of IPE cells isolated from rat and chicken irises were shown to demonstrate “stemness” [16]. A portion of purely isolated IPE cells of rodents, especially nestin-positive IPE cells, differentiated into multiple neuronal cell types, pan-neural marker- expressing cell types and retina-specific cell types without genetic manipulation. On the other hand, it has been shown that retinal stem cells are not present in the human iris [17], [18]. The present study demonstrates that human iris cells expressed stem cell markers such as nestin, N-cadherin, Sox2, Musashi-1 and Pax6. Expression of stem cell markers in iris cells may be attributed to the cell source, i.e. cells from infants. However, photoreceptor cell differentiation with exogenously added chemicals and growth factors was limited; that is, only green/red opsin was induced (Fig. 1). Other experimental evidence has also suggested the limitation in mammals without genetic manipulation. Progenitor cells from the mammalian iris, pars plana, and ciliary body do not show a convincing immunoreactivity for rhodopsin, phosducin, recoverin, PKC, or RPE65 [19], but are induced into photoreceptor progeny with retinal transcription factors [20], [21]. We first indicate that human IS cells that originate from neural crest (Fig. 3C), as well as IPE cells, differentiate into photoreceptor-like cells. Derivation of photoreceptor-like cells can be attributed to transgene-dependent differentiation of retinal progenitors that exist in the iris.

Our data show that induced photoreceptor-like cells have rod- and cone-signaling-pathways by RT-PCR and immunocytochemistry. In addition, expression of melanopsin was also detected in these cells. Photostimulation of the rod-or cone-pathway produces hyperpolarizing responses, while activation of the melanopsin-pathway produces depolarizing responses [22], [23], [24], [25], [26]. Melanopsin is intrinsically expressed in iris cells of the human (Fig. 4C), mouse [27] and Xenopus [24]. Melanopsin signaling has recently been reported to exist in both the iris and retina in mammals [27]. However, photostimulation did not produce any response in non-transfected human iris cells, suggesting the absence of phototransduction machineries *per se*. The light-induced depolarizing responses in infected cells indicate that phototransduction machinery for melanopsin-pathway was induced in infected cells. This is different from the results of infected monkey and rodent iris cells, where photostimulation produced hyperpolarizing responses [20]. Our data demonstrate that transduction of three transcriptional factors strongly induces expression of blue opsin, which suggests a potential to produce hyperpolarizing responses. One plausible reason for the differences is that numbers of expressed phototransduction machineries for rod-or cone-pathways are not enough in those cells, e.g. outer segments were not observed at an ultrastructural level. Although the reason for depolarization in transfected cells cannot be fully explained so far, it is likely that melanopsin-associated photoresponses may overcome cone- or rod- mediated photoresponses.

In addition to revealing insights into retinal transdifferentiation, this study describes the development of a novel experimental approach to genetic retinal diseases that may be relevant for beta cells, cardiomyocytes, and neurons [3], [4], [5]. Optimal donor cells for retinal transplantation are post-mitotic photoreceptor precursors but not mature photoreceptors [28]. Immature photoreceptors generated from ES cells or iPS cells in vitro differentiate through transplantation into the mouse retina [29], [30]. In this aspect, the induced retinal cells shown here may be suitable for future cell-based therapy since they are not fully differentiated. Together, the finding contributes substantially to an advance toward cell-based therapy for retinal genetic diseases.

## Materials and Methods

### Preparation of tissue and cell culture

Cells were obtained from donors at ages of 10 months, 1 year 8 months and 3 years. Iris tissues were excised from surgical specimens as a therapy for retinoblastoma with the approval of the Ethics Committee of the National Institute for Child and Health Development (NCCHD), Tokyo. The ethics committee of the NCCHD specifically approved this study (approval number, #156). Signed informed consent was obtained from the parents of the donors, and the surgical specimens were irreversibly de-identified. All experiments handling human cells and tissues were performed in line with the Tenets of the Declaration of Helsinki.

The iris was freed from the ciliary body. The iris kept away from a tumor and invasion of retinoblastoma cells were not detected by a pathologist's examination. The iris pieces were cut into smaller pieces and were subjected to explant-culture in the growth medium [Dulbecco's modified Eagle's medium (DMEM)/Nutrient mixture F12 (1∶1) supplemented with 10% fetal bovine serum, insulin-transferrin-selenium, and MEM-NEAA (GIBCO)]. Cells derived from the iris pieces were designated as “iris cells”. “IPE cells” were isolated from iris tissues using dispase and trypsin. “Iris cells” without “IPE cells” were designated as “iris-stromal (IS) cells” (Fig. 1A). Ciliary epithelial cells were isolated from pars plana and pars plicata in the same manner as IPE cells. Retinal pieces were cut into smaller pieces and were subjected to explant-culture in the same growth medium as iris cells. Second-passage cells were used for all the experiments.

### Sequencing of the RB gene

Total RNA was isolated from iris-derived cells of the three donors used in this study. An aliquot of total RNA was reverse transcribed into cDNA. The full-length of RB gene was amplified with Go-Taq polymerase (Promega) using the cDNA. Direct sequencing was performed with a BigDye® Terminator Cycle Sequencing Kit (Applied Biosystems, Foster City, CA). Sequencing reaction products were run on an automated capillary sequencer (Applied Biosystems 3130xl Genetic Analyzer; Applied Biosystems).

### Hanging drop method

Droplets, each of which included 1000 cells in 20 µl of culture medium, were formed on the inverted underside of a single Petri dish cover. The inverted bottom was then set on the top and the entire assembled Petri dish was re-inverted to its normal orientation. The drops of cell suspension were then hanging in the interior of the dish from the inner surface of the cover. The dishes were carefully placed into a 37°C incubator in an atmosphere of 5% CO_2_. Aggregates of cells were allowed to form in the drops for 24 h. Formed aggregates were then transferred to a Poly-D-lysine/laminin-coated 6-well tissue-culture plate (Becton Dickinson) (4 aggregates per well).

### Plasmid construction

Full length of transcription factors *SIX3* [31], *PAX6* [32], *RX* [33], *CRX* [34], *NRL* [35], [36] and *NEUROD* [10], were amplified from cDNAs prepared from total RNA of adult human retina (Clontech, CA, USA) by PCR, and cloned into the XmnI-EcoRV sites of pENTR11 (Invitrogen). Each vector contains one transcription factor and a mixture of vectors was used.

### Preparation and infection of recombinant retrovirus

The resulting pENTR11-transcription factors were recombined with pMXs-DEST by use of LR recombination reaction as instructed by the manufacturer (Invitrogen). pMXs was a gift from Dr. Kitamura (Tokyo University) and was modified into pMXs-DEST in our laboratory [37]. The retroviral DNAs were then transfected into 293FT cells and three days later the media were collected and concentrated. The iris-derived cells were prepared on laminin-coated six-well dishes or four-well chamber slides and maintained for one day. The cells were infected with above-mentioned media containing retroviral vector particles with 8 µg/ml of polybrene for 5 h at 37°C. After retroviral infection, the media were replaced with the DMEM/F12/B27 medium supplemented with 20 ng/ml bFGF, 40 ng/ml EGF, fibronectin, and 1% FBS. The retrovirus-infected cells were cultured for up to 21 days. We transfected retroviral eGFP under the same conditions to measure efficiency of infection. The frequency of eGFP-positive cells was 90–94% of all cells at 48 h after infection.

### Reverse transcriptase-PCR

Total RNA was isolated with an RNeasy Plus mini-kit® (Qiagen, Maryland, USA) or a PicoPure™ RNA Isolation Kit (Arcturus Bioscience, CA, USA) according to the manufacturer's instructions. An aliquot of total RNA was reverse transcribed using an oligo(dT) primer. The design of PCR primer sets is shown in Table 1.

**Table 1:** Primer sequences for RT-PCR.

Gene name	Forward	Reverse
Pax6	5′ – GTAGTTTCAGCACCAGTGTCTACC – 3′	5′ – GGCTGACTGTTCATGTGTGTCT – 3′
Rhodopsin	5′ – CAACTACATCCTGCTCAACCTAGC – 3′	5′ – GTGTAGTAGTCGATTCCACACGAG – 3′
Glutamine synthetase	5′ – GACCCTAACAAGCTGGTGTTATGT – 3′	5′ – ATGTACTTCAGACCATTCTCCTCC – 3′
CRALBP	5′ – GTCCTCTCTAGTCGGGACAAGTATG – 3′	5′ – CTGGTAGAAACCAGAAAGGTCATC – 3′
Recoverin	5′ – AGAGCATCTACGCCAAGTTCTTCC – 3′	5′ – GCAGAATTTCCTTATTGGCCAGTGTC – 3′
Peripherin	5′ – GTACCTGGCTATCTGTGTTCTCTTC – 3′	5′ – GTCGTAACTGTAGTGTGCTGAGTTG – 3′
Blue opsin	5′ – GCGCTACATTGTCATCTGTAAGCC – 3′	5′ – GAAGGAATGGTGACAAGCCGTAAG – 3′
Green/Red	5′ – GTGCAGTCTTACATGATTGTCCTC – 3′	5′ – AGATAACGGGGTTGTAGATAGTGG – 3′
Green	5′ – GTGATGGTCCTGGCATTC – 3′	5′ – GAGGACACAGATGAGACCTCCGTT – 3′
Red	5′ – GTGATGATCTTTGCGTAC – 3′	5′ – GAGGACACAGATGAGACCTCCGTT – 3′
Transducin-α2-chain	5′ – ATTACAGACCCTGAGTACCTCCCTA – 3′	5′ – GAGGTCCTTCTTGTTGAGAAAGAG – 3′
Cone channel A3 (CNGA3)	5′ – GTCCTGTATGTCTTGGATGTGC – 3′	5′ – GAATCAATCTTGGCCTGGAACTCTG – 3′
Transducin	5′ – CATCGAGACGCAGTTCTCCT – 3′	5′ – AGTAGCGGTGGTTGCAGATG – 3′
Phosducin	5′ – TCAAAGGAACGAGTCAGCAG – 3′	5′ – CTGCTGCAAGGCATGTTAAA – 3′
PDE6b	5′ – CAGTGATGAACACCGACACC – 3′	5′ – ATTTGACCAGGTCCAGTTCG – 3′
PDE6c	5′ – CTGAGGTGGCCTCTAGGTTG – 3′	5′ – GCTGGTGTGATGAAGCCTTAG – 3′
Rhodopsin kinase (GRK1)	5′ – GGACTGGTTCCTGGACTTCA – 3′	5′ – AAGCCAGGGTTCTCCTCATT – 3′
S-antigen	5′ – GGTGTTGTCCTGGTTGATCC – 3′	5′ – TCAGCGTCTTGGTCAAAGTG – 3′
Arrestin3 (ARR3)	5′ – GGTGTTGTCCTGGTTGATCC – 3′	5′ – GTCACAGAACAGGGCAGGTT – 3′
Retinol dehydrogenase 12 (RDH12)	5′– CTTCTCCCCCTTTGTCAAGA – 3′	5′ – CTTTAGGGTTGGCCTTCTCC – 3′
GFAP	5′– GATCAACTCACCGCCAACAG – 3′	5′ – GGACGCCATTGCCTCATACTG – 3′
Nurr1	5′– TTTCTGCCTTCTCCTGCATT – 3′	5′ – GTGGCACCAAGTCTTCCAAT – 3′
Nestin	5′ – AGAGGGGAATTCCTGGAG – 3′	5′ – CTGAGGACCAGGACTCTCTA – 3′
NF-M	5′ – TGAGCTACACGTTGGACTCG – 3′	5′ – TCTCCGCCTCAATCTCCTTA – 3′
Sox-2	5′ – CACAACTCGGAGATCAGCAA – 3′	5′ – GTTCATGTGCGCGTAACTGT – 3′
MAP-2	5′ – GGATTCTGGCAGCAGTTCTC – 3′	5′ – TCCTTGCAGACACCTCCTCT – 3′
Musashi1	5′ – CGAGCTTACAGCCATTCCTC – 3′	5′ – ACTCGTGGTCCTCAGTCAGC – 3′
Tyrosine hydroxylase	5′ – GTCCCGAGCTGTGAAGGTGTTTGA – 3′	5′ – ATTGTCTTCCCGGTAGCCGCTGAA – 3′
Twist	5′ – GTCCGCAGTCTTACGAGGAG – 3′	5′ – GCTTGAGGGTCTGAATCTTGCT – 3′
Snail	5′ – AATCGGAAGCCTAACTACAGCG – 3′	5′ – GTCCCAGATGAGCATTGGCA – 3′
Slug	5′ – AAGCATTTCAACGCCTCCAAA – 3′	5′ – AGGATCTCTGGTTGTGGTATGAC – 3′
Sox9	5′ – AGACAGCCCCCTATCGACTTC – 3′	5′ – TGCTGCTTGGACATCCACAC – 3′
P75NTR	5′ – CCTACGGCTACTACCAGGATG – 3′	5′ – CACACGGTGTTCTGCTTGTC – 3′
Melanopsin	5′ – CTTCACCAGTAGCCTCTATAAGCAG – 3′	5′ – CCCTGAAGATGAAGATGTAGCAGT – 3′
G3PDH	5′ – GCTCAGACACCATGGGGAAGGT – 3′	5′ – GTGGTGCAGGAGGCATTGCTGA – 3′

### Quantitative RT-PCR

The cDNA templates were amplified (ABI7900HT Sequence Detection System) using the Platinum Quantitative PCR SuperMix-UDG with ROX (11743-100, Invitrogen). Fluorescence was monitored during every PCR cycle at the annealing step. The authenticity and size of the PCR products were confirmed using a melting curve analysis (using software provided by Applied Biosystems) and a gel analysis. mRNA levels were normalized using G3PDH as a housekeeping gene. The design of PCR primer sets is shown in Table 2.

**Table 2:** Primer sequences for qRT-PCR.

Gene name	Forward	Reverse
Recoverin	5′ – TTCAAGGAGTACGTCATCGCC – 3′	5′ – GATGGTCCCGTTACCGTCC – 3′
S-arrestin	5′ – GGACAAATCGGTGACCATCTAC – 3′	5′ – ACAGGAGGATACACCTGGACC – 3′
Phosphodiesterase 6B	5′ – ACGTGTGGTCTGTGCTGATG – 3′	5′ – CTTGCCGTGGAGGATGTAGTC – 3′
Rhodopsin	5′ – CACCTCTCTGCATGGATACTTCG – 3′	5′ – ATGGGCTTACACACCACCAC – 3′
Blue opsin	5′ – TAGCAGGTCTGGTTACAGGATG – 3′	5′ – GAGACGCCAATACCAATGGTC – 3′
Green opsin	5′ – CATCCGCAGGACAGCTATGAG – 3′	5′ – GTAAGCACAGTGGGTTCGTTTCCC – 3′
Phosphodiesterase 6C	5′ – AGGCTTCATCACACCAGCTAC – 3′	5′ – TGAAACTGTCGCTCAACATCTG – 3′
Cone channel A3	5′ – GGACTCTTTTCCTGATCGTTTCC – 3′	5′ – GCTGGTGTTAGTGTTGCATTTG – 3′
Cone channel B3	5′ – CTCCTGTGGCTCTTGCTTGTC – 3′	5′ – GCGGTTTGATATGGGAAGACGA – 3′
Arrestin3	5′ – GCACAAGCTAGGGGACAATG – 3′	5′ – CCAGCCGCACATAGTCTCTC – 3′
G3PDH	5′ – GCTCAGACACCATGGGGAAGGT – 3′	5′ – GTGGTGCAGGAGGCATTGCTGA – 3′

### Immunocytochemistry

Immunocytochemical analysis was performed as previously described [38]. As a methodological control, the primary antibody was omitted. The primary and secondary antibodies used were as follows: blue opsin (rabbit polyclonal, H-40, Santa Cruz), green/red opsin (goat polyclonal, C-19, Santa Cruz), rhodopsin (goat polyclonal, I-17, Santa Cruz), N-Cadherin (rabbit polyclonal, Abcam), GFAP (rabbit polyclonal, DAKO), NSE (mouse monoclonal, VI-H14, DAKO), Vimentin (mouse monoclonal, V9, DAKO), Nestin (mouse monoclonal, clone196908, R&D), Sox2 (rabbit polyclonal, ab15830, Abcam), melanopsin (goat polyclonal, C-16, Santa Cruz), recoverin (mouse monoclonal, 6A55CD6, Santa Cruz).

### Light stimulation

A high pressure UV lamp (USH-102D, Ushio) was used as a light source. Diffuse, unpolarized blue and green lights were generated through bandpass filters attached with the fluorescent emission system (BX-FLA, Olympus, Tokyo, Japan). Wavelength of light for stimulation was 460–490 nm for blue and 520–550 nm for green. Duration and timing of light stimulation was monitored by a photodiode (TPS708, Toshiba). Light intensity was calibrated by a light meter (LI-COR, LI-250) that was placed at the focal plane on the stage. To maximize a chance for photoisomelization of photopigment, we applied a strong light to the cell. Light intensity used for stimulation was 390 W/m^2^ for blue and 4810 W/m^2^ for green.

### Electrophysiology

To activate the phototransduction cascade, 11-cis retinal (a gift from the vision research community, the National Eye Institute, National Institutes of Health) was added to the culture medium of the human iris-derived cells to a concentration of 50 µM with 0.2% ethanol as a vehicle, approximately 2 h prior to the electrical recording. The cells were kept at 37°C in the dark and were transferred to a recording chamber filled with Leibovitz's L-15 medium (Gibco) and mounted on the microscope stage (BX51WI; Olympus, Tokyo, Japan) under dim red light. Individual cells were visualized under an infrared light monitoring system. Electrical recordings were made in the whole-cell patch-clamp configuration. Patch pipettes were pulled from borosilicate glass (Hilgenberg GmbH, Marsfeld, Germany) using a two-stage electrode puller (PP-83; Narishige, Tokyo, Japan). The composition of the intra-pipette solution was (in mM) KCl, 135; CaCl_2_, 0.5; HEPES, 5; EGTA, 5; ATP-2Na, 5; GTP-3Na, 1; and pH was adjusted to 7.3 with KOH. The resistance of patch pipettes was 12–15 MΩ when filled with an intra-pipette solution. An Ag-AgCl pellet submerged in a NaCl well and connected to a recording chamber via a 150 mM NaCl agar-bridge was used as a reference electrode. The membrane current was recorded with a patch-clamp amplifier (Axopatch-200B; Axon Instruments, Foster City, CA, USA), low-pass filtered with a cutoff frequency of 500 Hz, and digitized at 1 kHz through a DigiData 1322A Interface using pCLAMP software (version 8.0, Axon Instruments).

To assess whether a recorded cell had any response to light or not, we used the following criteria:
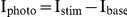
Where I_base_ was an average of holding current for 1 s just before light stimulation and I_stim_ was an average of holding current for 1 s just before the cessation of light stimulation. When I_photo_ was larger than the two times of standard deviation of I_base_, I_photo_ was judged as a real response to light stimulation.

## Supporting Information

Figure S1 **Southern blot analysis.** A. Genomic DNA was isolated using the DNeasy kit (Qiagen). Genomic DNA (500 ng) was digested with BamHI restriction enzyme, separated via 0.8% agarose gel electrophoresis, and transferred to Hybond-N membranes (GE Healthcare). The membrane was then fixed under UV irradiation. The full-length RB gene probe was labeled by the AlkPhos Direct Labelling Reagent (GE Healthcare) and hybridized to the blot and detected using CDP-Star detection reagent (GE Healthcare). Lane 1: iris-derived cells (EY1420), lane 2: iris-derived cells (EY1406), lane 3: iris-derived cells (EY1408), lane 4: menstrual blood-derived cells (control), lane 5: endometrium-derived cells (control). B. Ethidium bromide stain of the BamHI-digested genomic DNA after electrophoresis.
(DOC)

Figure S2 **RT-PCR analysis for genes of MAP2, rhodopsin, blue opsin, green/red opsin and G3PDH in iris-derived cells after gene transduction of several transcription factors.** As negative controls, data of iris tissue and cultured iris-derived cells without gene transduction (w/o) are shown. We selected six genes, SIX3, PAX6, RX, CRX, NRL, and NEUROD, as candidate factors that may contribute to induce photoreceptor-specific phenotypes in iris cells. SIX3, PAX6, RX, CRX, NRL, and NEUROD are indicated as S, P, R, C, NR and ND, respectively. Left panel: Transduction of each single gene of SIX3, PAX6, RX, CRX, NRL, or NEUROD. Right panel: Transduction of all six genes and 5 genes. To determine which of the six candidates are critical, we examined the effect of withdrawal of individual factors from the pool of the candidate genes on expression of the opsin genes. As a result, individual withdrawal of NEUROD resulted in loss of expression of rhodopsin and withdrawal of CRX resulted in loss of blue opsin.
(DOC)

Figure S3 **Immunocytochemistry using antibodies to rhodopsin (left panels), blue opsin (middle panels) and green/red opsin (right panels) on the cultured iris cells without gene infection (upper panels) and frozen sections of human retina (lower panels).** Human iris cells without gene infection (upper panels) and the macular area of human retina (lower panels) served negative and positive controls, respectively, for Fig. 2C. The primary antibodies used were as follows: rhodopsin (goat polyclonal, I-17, Santa Cruz), blue opsin (goat polyclonal, P-13, Santa Cruz), and green/red opsin (goat polyclonal, C-19, Santa Cruz). The secondary antibody used was rabbit polyclonal to goat IgG conjugated with FITC. Nuclei were stained with DAPI. The photoreceptor layer in the retina is positive for rhodopsin, blue opsin and green/red opsin (lower panels from left to right).
(DOC)

Figure S4 **Immunocytochemistry using antibodies to blue opsin (red) and rhodopsin (green).** Double-stained immunocytochemistry was performed, by using 2 primary antibodies (mouse monoclonal Ab to rhodopsin and rabbit polyclonal Ab to blue opsin) and 2 secondary antibodies (goat anti-mouse polyclonal FITC-labeled Ab and goat anti-rabbit polyclonal rhodamine-labeled Ab). Nuclei were stained with DAPI (blue). Experiments were performed at two weeks after infection. Scale bar represents 50 µm in the rightmost panel.
(DOC)

Figure S5 **Electron-microscopic observation.** Cells were initially fixed in PBS containing 2.5% glutaraldehyde for 24 h, and were embedded in epoxy resin. Ultrathin sections were double-stained with uranyl acetate and lead citrate, and were viewed under a JEM-1200EX transmission electron microscope (JEOL, Ltd.). After transduction of the RX, CRX, and NEUROD genes into human cultured iris cells, a cilia-associated structure, i.e. centriole (arrow head) surrounded by mitochondria (arrows), was detected.
(DOC)

Figure S6 **Quantitative analysis of photoreceptor-specific/associated genes expression (blue opsin, s-antigen and recoverin).** Individual RNA expression levels were normalized by respective G3PDH expression levels. Vertical axis is relative expression levels of each gene in the siCRX & siNEUROD-transfected cells versus negative control siRNA-transfected cells (%, the mean of relative expression levels of two experiments). **siRNA transfection.** The cells at 7 days after transduction of the CRX, RX, and NEUROD genes in 6 well plates were transfected with siRNA using Lipofectamine RNAiMAX Reagent (Invitrogen) according to the protocols recommended by the manufacturer. The cells were harvested 48 h after transfection and analyzed by quantitative RT-PCR. Stealth RNAiTM siRNA Duplex Oligoribonucleotides (siCRX and siNEUROD1, Invitrogen) were used as siRNAs to the CRX and NEUROD genes, and StealthTM RNAi Negative Control Duplexes (Invitrogen) were used as control siRNA.
(DOC)

Table S1 **Primer sequences for exogenous/endogenous expression of transcription factors.**
(DOC)

Table S2 **Opsin expression by combination of transcription factors.**
(DOC)
