# Supplementary material for: Derivation of Human Differential Photoreceptor-like Cells from the Iris by Defined Combinations of CRX, RX and NEUROD
Source: PLoS One. 2012 Apr 25;7(4):e35611. doi: 10.1371/journal.pone.0035611 (PMC3338414; doi:10.1371/journal.pone.0035611)
Supplement: Figure S1 — Southern blot analysis. A. Genomic DNA was isolated using the DNeasy kit (Qiagen). Genomic DNA (500 ng) was digested with BamHI restriction enzyme, separated via 0.8% agarose gel electrophoresis, and transferred to Hybond-N membranes (GE Healthcare). The membrane was then fixed under UV irradiation. The full-length RB gene probe was labeled by the AlkPhos Direct Labelling Reagent (GE Healthcare) and hybridized to the blot and detected using CDP-Star detection reagent (GE Healthcare). Lane 1: iris-derived cells (EY1420), lane 2: iris-derived cells (EY1406), lane 3: iris-derived cells (EY1408), lane 4: menstrual blood-derived cells (control), lane 5: endometrium-derived cells (control). B. Ethidium bromide stain of the BamHI-digested genomic DNA after electrophoresis. (DOC) [file pone.0035611.s001.doc]

**Figure S1**


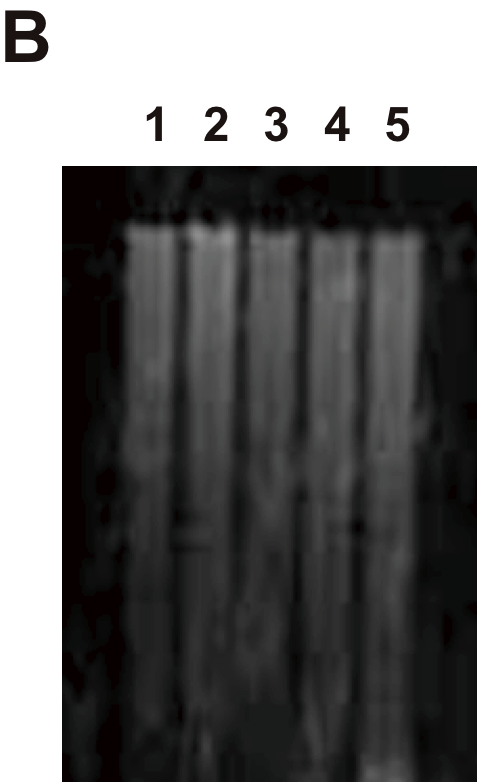

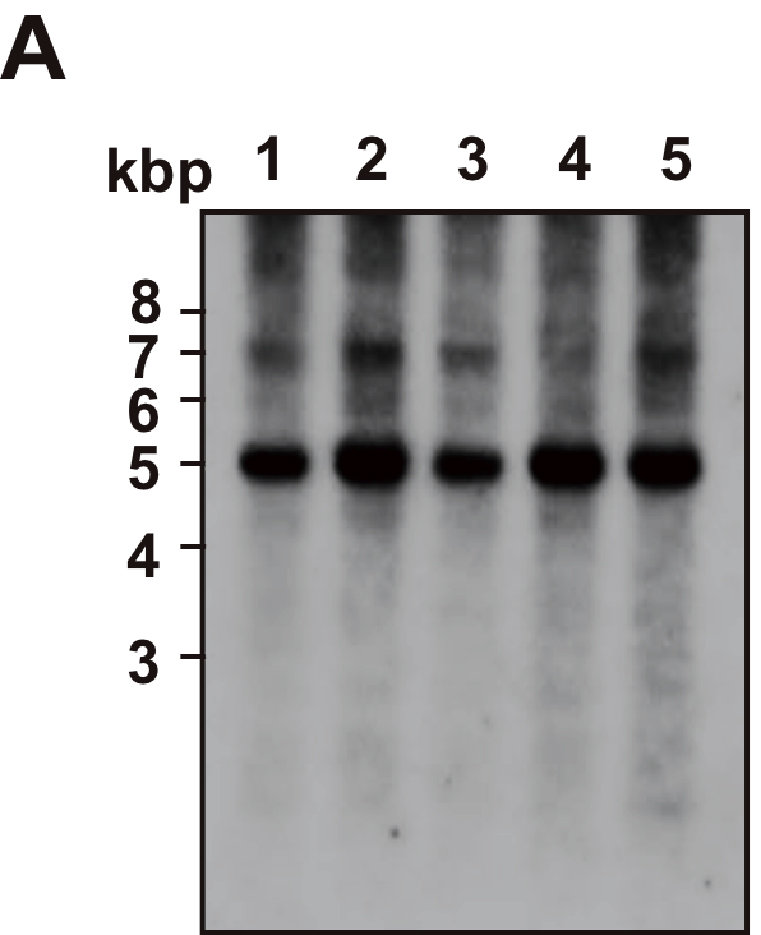


**Fig. S1. Southern blot analysis**

A. Genomic DNA was isolated using the DNeasy kit (Qiagen). Genomic DNA (500 ng) was digested with BamHI restriction enzyme, separated via 0.8% agarose gel electrophoresis, and transferred to Hybond-N membranes (GE Healthcare). The membrane was then fixed under UV irradiation. The full-length RB gene probe was labeled by the AlkPhos Direct Labelling Reagent (GE Healthcare) and hybridized to the blot and detected using CDP-Star detection reagent (GE Healthcare).

Lane 1: iris-derived cells (EY1420), lane 2: iris-derived cells (EY1406), lane 3: iris-derived cells (EY1408), lane 4: menstrual blood-derived cells (control), lane 5: endometrium-derived cells (control).

B. Ethidium bromide stain of the BamHI-digested genomic DNA after electrophoresis.
