# Supplementary material for: Derivation of Human Differential Photoreceptor-like Cells from the Iris by Defined Combinations of CRX, RX and NEUROD
Source: PLoS One. 2012 Apr 25;7(4):e35611. doi: 10.1371/journal.pone.0035611 (PMC3338414; doi:10.1371/journal.pone.0035611)
Supplement: Figure S2 — RT-PCR analysis for genes of MAP2, rhodopsin, blue opsin, green/red opsin and G3PDH in iris-derived cells after gene transduction of several transcription factors. As negative controls, data of iris tissue and cultured iris-derived cells without gene transduction (w/o) are shown. We selected six genes, SIX3, PAX6, RX, CRX, NRL, and NEUROD, as candidate factors that may contribute to induce photoreceptor-specific phenotypes in iris cells. SIX3, PAX6, RX, CRX, NRL, and NEUROD are indicated as S, P, R, C, NR and ND, respectively. Left panel: Transduction of each single gene of SIX3, PAX6, RX, CRX, NRL, or NEUROD. Right panel: Transduction of all six genes and 5 genes. To determine which of the six candidates are critical, we examined the effect of withdrawal of individual factors from the pool of the candidate genes on expression of the opsin genes. As a result, individual withdrawal of NEUROD resulted in loss of expression of rhodopsin and withdrawal of CRX resulted in loss of blue opsin. (DOC) [file pone.0035611.s002.doc]

**Figure S2**


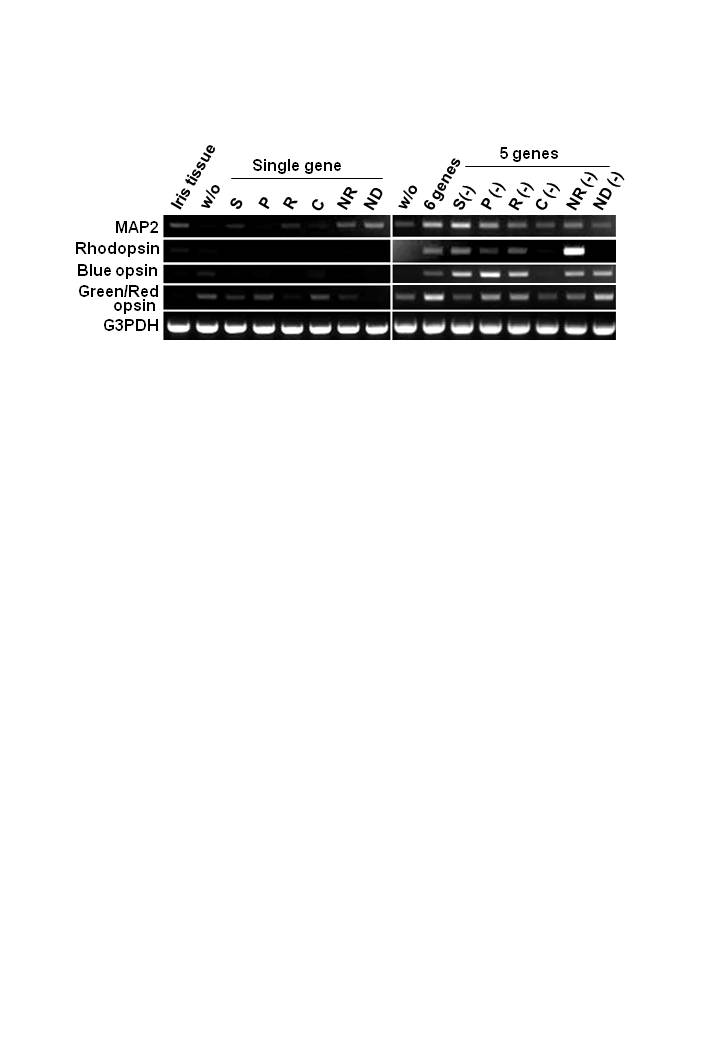


**Fig. S2. RT-PCR analysis for genes of MAP2, rhodopsin, blue opsin, green/red opsin and G3PDH in iris-derived cells after gene transduction of several transcription factors.** As negative controls, data of iris tissue and cultured iris-derived cells without gene transduction (w/o) are shown. We selected six genes, SIX3, PAX6, RX, CRX, NRL, and NEUROD, as candidate factors that may contribute to induce photoreceptor-specific phenotypes in iris cells. SIX3, PAX6, RX, CRX, NRL, and NEUROD are indicated as S, P, R, C, NR and ND, respectively. Left panel: Transduction of each single gene of SIX3, PAX6, RX, CRX, NRL, or NEUROD. Right panel: Transduction of all six genes and 5 genes. To determine which of the six candidates are critical, we examined the effect of withdrawal of individual factors from the pool of the candidate genes on expression of the opsin genes. As a result, individual withdrawal of NEUROD resulted in loss of expression of rhodopsin and withdrawal of CRX resulted in loss of blue opsin.
