# Supplementary material for: Derivation of Human Differential Photoreceptor-like Cells from the Iris by Defined Combinations of CRX, RX and NEUROD
Source: PLoS One. 2012 Apr 25;7(4):e35611. doi: 10.1371/journal.pone.0035611 (PMC3338414; doi:10.1371/journal.pone.0035611)
Supplement: Figure S3 — Immunocytochemistry using antibodies to rhodopsin (left panels), blue opsin (middle panels) and green/red opsin (right panels) on the cultured iris cells without gene infection (upper panels) and frozen sections of human retina (lower panels). Human iris cells without gene infection (upper panels) and the macular area of human retina (lower panels) served negative and positive controls, respectively, for Fig. 2C. The primary antibodies used were as follows: rhodopsin (goat polyclonal, I-17, Santa Cruz), blue opsin (goat polyclonal, P-13, Santa Cruz), and green/red opsin (goat polyclonal, C-19, Santa Cruz). The secondary antibody used was rabbit polyclonal to goat IgG conjugated with FITC. Nuclei were stained with DAPI. The photoreceptor layer in the retina is positive for rhodopsin, blue opsin and green/red opsin (lower panels from left to right). (DOC) [file pone.0035611.s003.doc]

**Figure S3**

**
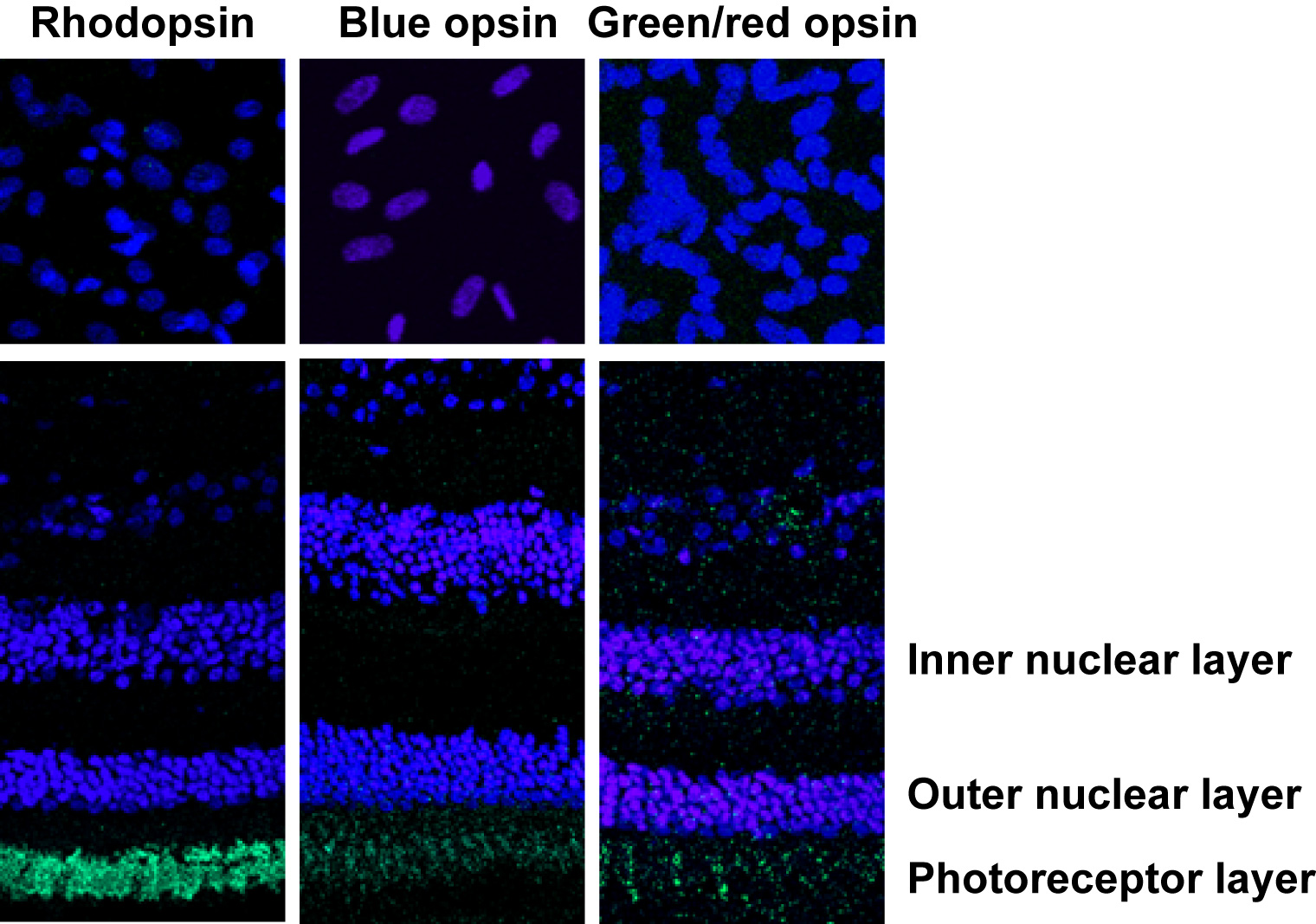
**

**Fig. S3. Immunocytochemistry using antibodies to rhodopsin (left panels), blue opsin (middle panels) and green/red opsin (right panels) on the cultured iris cells without gene infection (upper panels) and frozen sections of human retina (lower panels).** Human iris cells without gene infection (upper panels) and the macular area of human retina (lower panels) served negative and positive controls, respectively, for Fig. 2C. The primary antibodies used were as follows: rhodopsin (goat polyclonal, I-17, Santa Cruz), blue opsin (goat polyclonal, P-13, Santa Cruz), and green/red opsin (goat polyclonal, C-19, Santa Cruz). The secondary antibody used was rabbit polyclonal to goat IgG conjugated with FITC. Nuclei were stained with DAPI. The photoreceptor layer in the retina is positive for rhodopsin, blue opsin and green/red opsin (lower panels from left to right).

were used as control siRNA.
