# Supplementary material for: Derivation of Human Differential Photoreceptor-like Cells from the Iris by Defined Combinations of CRX, RX and NEUROD
Source: PLoS One. 2012 Apr 25;7(4):e35611. doi: 10.1371/journal.pone.0035611 (PMC3338414; doi:10.1371/journal.pone.0035611)
Supplement: Figure S4 — Immunocytochemistry using antibodies to blue opsin (red) and rhodopsin (green). Double-stained immunocytochemistry was performed, by using 2 primary antibodies (mouse monoclonal Ab to rhodopsin and rabbit polyclonal Ab to blue opsin) and 2 secondary antibodies (goat anti-mouse polyclonal FITC-labeled Ab and goat anti-rabbit polyclonal rhodamine-labeled Ab). Nuclei were stained with DAPI (blue). Experiments were performed at two weeks after infection. Scale bar represents 50 µm in the rightmost panel. (DOC) [file pone.0035611.s004.doc]

**Figure S4**

**
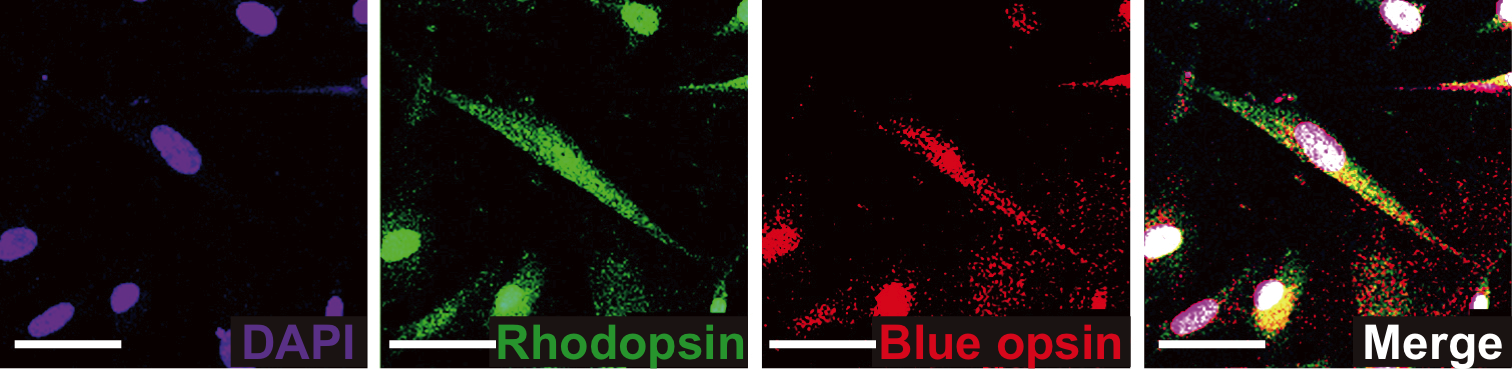
**

**Fig. S4. Immunocytochemistry using antibodies to blue opsin (red) and rhodopsin (green).**

Double-stained immunocytochemistry was performed, by using 2 primary antibodies (mouse monoclonal Ab to rhodopsin and rabbit polyclonal Ab to blue opsin) and 2 secondary antibodies (goat anti-mouse polyclonal FITC-labeled Ab and goat anti-rabbit polyclonal rhodamine-labeled Ab). Nuclei were stained with DAPI (blue). Experiments were performed at two weeks after infection. Scale bar represents 50 μm in the rightmost panel.
