# Supplementary material for: Derivation of Human Differential Photoreceptor-like Cells from the Iris by Defined Combinations of CRX, RX and NEUROD
Source: PLoS One. 2012 Apr 25;7(4):e35611. doi: 10.1371/journal.pone.0035611 (PMC3338414; doi:10.1371/journal.pone.0035611)
Supplement: Figure S5 — Electron-microscopic observation. Cells were initially fixed in PBS containing 2.5% glutaraldehyde for 24 h, and were embedded in epoxy resin. Ultrathin sections were double-stained with uranyl acetate and lead citrate, and were viewed under a JEM-1200EX transmission electron microscope (JEOL, Ltd.). After transduction of the RX, CRX, and NEUROD genes into human cultured iris cells, a cilia-associated structure, i.e. centriole (arrow head) surrounded by mitochondria (arrows), was detected. (DOC) [file pone.0035611.s005.doc]

**Figure S5**

**
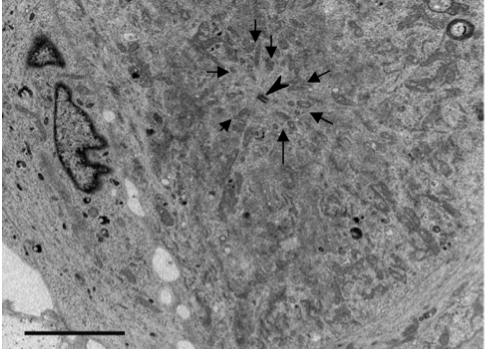
**

**Fig. S5. Electron-microscopic observation**

Cells were initially fixed in PBS containing 2.5% glutaraldehyde for 24 h, and were embedded in epoxy resin. Ultrathin sections were double-stained with uranyl acetate and lead citrate, and were viewed under a JEM-1200EX transmission electron microscope (JEOL, Ltd.). After transduction of the *RX, CRX,* and *NEUROD* genes into human cultured iris cells, a cilia-associated structure, i.e. centriole (arrow head) surrounded by mitochondria (arrows), was detected.
