# Supplementary material for: Derivation of Human Differential Photoreceptor-like Cells from the Iris by Defined Combinations of CRX, RX and NEUROD
Source: PLoS One. 2012 Apr 25;7(4):e35611. doi: 10.1371/journal.pone.0035611 (PMC3338414; doi:10.1371/journal.pone.0035611)
Supplement: Figure S6 — Quantitative analysis of photoreceptor-specific/associated genes expression (blue opsin, s-antigen and recoverin). Individual RNA expression levels were normalized by respective G3PDH expression levels. Vertical axis is relative expression levels of each gene in the siCRX & siNEUROD-transfected cells versus negative control siRNA-transfected cells (%, the mean of relative expression levels of two experiments). siRNA transfection. The cells at 7 days after transduction of the CRX, RX, and NEUROD genes in 6 well plates were transfected with siRNA using Lipofectamine RNAiMAX Reagent (Invitrogen) according to the protocols recommended by the manufacturer. The cells were harvested 48 h after transfection and analyzed by quantitative RT-PCR. Stealth RNAiTM siRNA Duplex Oligoribonucleotides (siCRX and siNEUROD1, Invitrogen) were used as siRNAs to the CRX and NEUROD genes, and StealthTM RNAi Negative Control Duplexes (Invitrogen) were used as control siRNA. (DOC) [file pone.0035611.s006.doc]

**Figure S6**

**
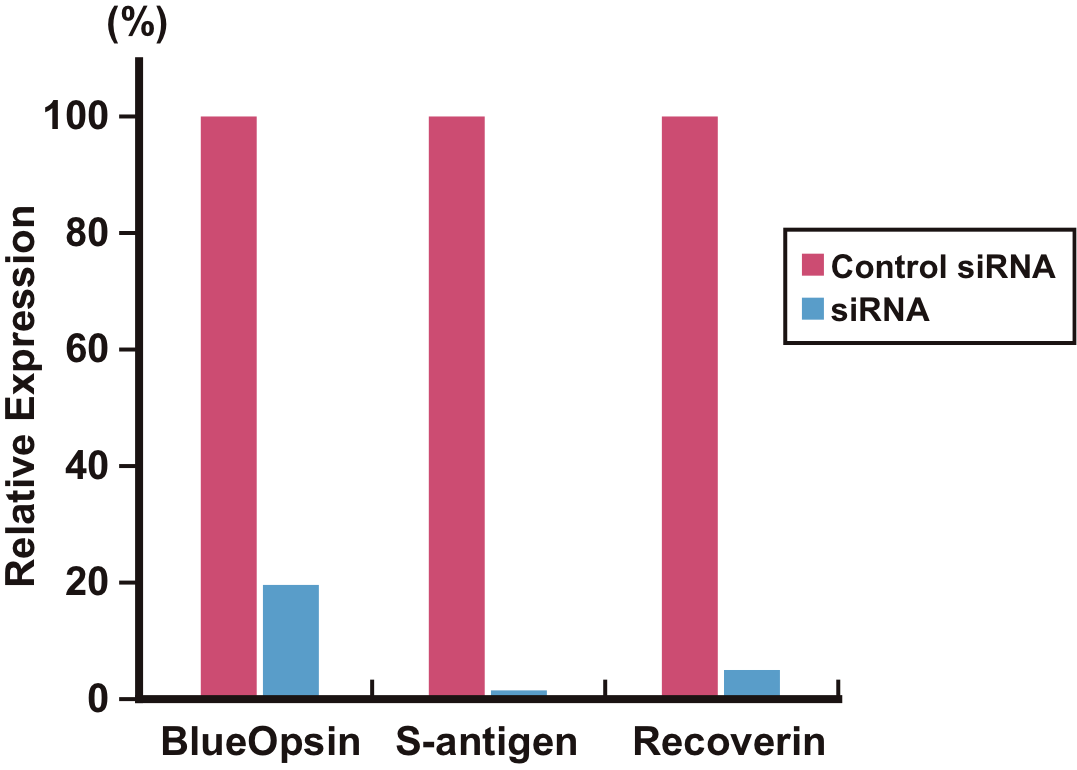
**

**Fig. S6. Quantitative analysis of photoreceptor-specific/associated genes expression (blue opsin, s-antigen and recoverin).**

Individual RNA expression levels were normalized by respective *G3PDH* expression levels. Vertical axis is relative expression levels of each gene in the siCRX & siNEUROD-transfected cells versus negative control siRNA-transfected cells (%, the mean of relative expression levels of two experiments).

**siRNA transfection**

The cells at 7 days after transduction of the *CRX, RX*, and *NEUROD* genes in 6 well plates were transfected with siRNA using Lipofectamine RNAiMAX Reagent (Invitrogen) according to the protocols recommended by the manufacturer. The cells were harvested 48 h after transfection and analyzed by quantitative RT-PCR. Stealth RNAi^TM^ siRNA Duplex Oligoribonucleotides (siCRX and siNEUROD1, Invitrogen) were used as siRNAs to the *CRX* and *NEUROD* genes, and Stealth^TM^ RNAi Negative Control Duplexes (Invitrogen) were used as control siRNA.
