# Supplementary material for: Derivation of Human Differential Photoreceptor-like Cells from the Iris by Defined Combinations of CRX, RX and NEUROD
Source: PLoS One. 2012 Apr 25;7(4):e35611. doi: 10.1371/journal.pone.0035611 (PMC3338414; doi:10.1371/journal.pone.0035611)
Supplement: Table S1 — Primer sequences for exogenous/endogenous expression of transcription factors. (DOC) [file pone.0035611.s007.doc]

**Table S1**

**Primer sequences for exogenous/endogenous expression of transcription factors**

Gene name Forward Reverse

Endogenous Crx 5’ –CCAGGCTTAAAATCTCCCCATGT – 3’ 5’ –CCGGTTCTTGAACCAAACCTGAA – 3’

Endogenous NeuroD 5’ –GCGCTTAGCATCACTAACTGG – 3’ 5’ –GTCTCTTGGGCTTTTGATCGT – 3’

Endogenous Rx 5’ –GTCTGAAAGCCAAGGAGCACA – 3’ 5’ –CTCCCGTACCCCAATATTCACTC – 3’

Exogenous Crx 5’ –AGTAGACGGCATCGCAGCTTG– 3’ 5’ –CCGGTTCTTGAACCAAACCTGAA – 3’

Exogenous NeuroD 5’ –AGTAGACGGCATCGCAGCTTG– 3’ 5’ –GTCTCTTGGGCTTTTGATCGT – 3’

Exogenous Rx 5’ –AGTAGACGGCATCGCAGCTTG– 3’’ 5’ –CTGCAGCTTCATGGAGGACAC – 3’
