# Supplementary material for: Derivation of Human Differential Photoreceptor-like Cells from the Iris by Defined Combinations of CRX, RX and NEUROD
Source: PLoS One. 2012 Apr 25;7(4):e35611. doi: 10.1371/journal.pone.0035611 (PMC3338414; doi:10.1371/journal.pone.0035611)
Supplement: Table S2 — Opsin expression by combination of transcription factors. (DOC) [file pone.0035611.s008.doc]

**Table S2**

**Opsin expression by combination of transcription factors**

| Gene set | Rhodopsin | Blue opsin | Green/Red opsin |
| --- | --- | --- | --- |
| *CRX & RX* | **-** | **+** | **+** |
| *CRX & NEUROD* | **+** | **+** | **-** |
| *CRX & RX & NEUROD* | **+** | **++** | **±** |
